# Supplementary material for: PPM1M, a LRRK2-counteracting, phosphoRab12-preferring phosphatase with potential link to Parkinson’s disease
Source: bioRxiv. 2025 Mar 19:2025.03.19.644182. Preprint. [Version 1] doi: 10.1101/2025.03.19.644182 (PMC11957146; doi:10.1101/2025.03.19.644182)
Supplement: 1 [file NIHPP2025.03.19.644182V1-supplement-1.pdf]

bioRxiv preprint doi: https://doi.org/10.1101/2025.03.19.644182; this version posted March 19, 2025. The copyright holder for this preprint (which was not certified by peer review) is the author/funder, who has granted bioRxiv a license to display the preprint in perpetuity. It is made available under aCC-BY 4.0 International license.

Supplemental Materials

Supplemental Figure 1. Immunoblots used for screen quantitation. Each lane represents a unique siRNA from the library or Rab12 siRNA, non-targeting siRNA, or non-targeting siRNA with MLi-2. siRNAs were identified by ID number only (labeled below each blot) during screen and analysis. A list of all genes and guides is included in Supplemental Table 2.

Supplemental Figure 2. Quantitative analysis of the phosphatome-wide screen. Quantitation of pRab12/total Rab12 and pRab10/total Rab10 from gels in Supplemental Figure 1, normalized to non-targeting (NT) control. Values are the average of two independent analyses of the same data.

Supplemental Figure 3. PPM1M knockdown in 3T3 and MEF cells increases pRab12 levels. (A) Immunoblot analysis of 3T3 and MEF cells treated with non-targeting (NT) or PPM1M siRNA for 72h, followed by 200 nM MLi-2 for 20 minutes as indicated. **(B)** Quantitation of pRab12 levels from immunoblots in (A) normalized to respective NT controls. Error bars indicate SEM from three independent experiments carried out in duplicate. Statistical significance determined by student's T-test, respective to NT. **p=0.0093 for 3T3, ****p<0.0001 for MEF. **(C)** Genotyping results of pooled CRISPR knockouts for *Ppm1h*, *Ppm1m*, and *Ppm1j* MEFs by Synthego ICE software. For each cell line, guide sequences, indel frequency (%), and sequencing traces for edited and control samples for each guide are shown.

Supplemental Figure 4. (A) SDS-PAGE elution profiles for His-SUMO-PPM1M after purification on a Superdex 200 16/60 120 mL column (top row) and SUMO-tag cleaved (His-SUMO-)Rab10 (middle panel) or (His-SUMO-)Rab12 (bottom panel) after purification on a Superdex 200 10/300 24 mL column. Fraction 72 was used for His-SUMO-PPM1M in experiments. Mass is shown at right in kDa. **(B)** PPM1M chromatographs as a dimer in cytosol. HEK293 cytosol overexpressing HA-PPM1M was resolved on a Superdex 200 10/300 24 mL FPLC column. Immunoblot of fractions collected and their quantitation is shown.

Supplemental Table 1. Key Resource Table

Supplemental Table 2. List of siRNA targets and sequences of all siRNA oligonucleotides used in phosphatome-wide siRNA screen. 4 siRNAs per gene were provided in SMARTpool format. Of note, the Dharmacon commercial mouse phosphatase library did not include all phosphatase genes; we also created a custom library of 89 additional phosphatase genes. These are described respectively as G-113705 Lot 23107 (commercial library) or "cherry pick" library in the table. During siRNA screen and analysis, samples were identified only by the gene ID numbers listed.

Supplemental Table 3. Overview of cohorts that were interrogated for *PPM1M* p.D440N carrier status. Included is the respective cohort including total number of individuals per study, number of disease and control subjects, number of individuals carrying the *PPM1M* D440N variant in the heterozygous state with and without PD, and minor allele frequency (MAF). YOPD = young onset PD.

bioRxiv preprint doi: https://doi.org/10.1101/2025.03.19.644182; this version posted March 19, 2025. The copyright holder for this preprint (which was not certified by peer review) is the author/funder, who has granted bioRxiv a license to display the preprint in perpetuity. It is made available under aCC-BY 4.0 International license.

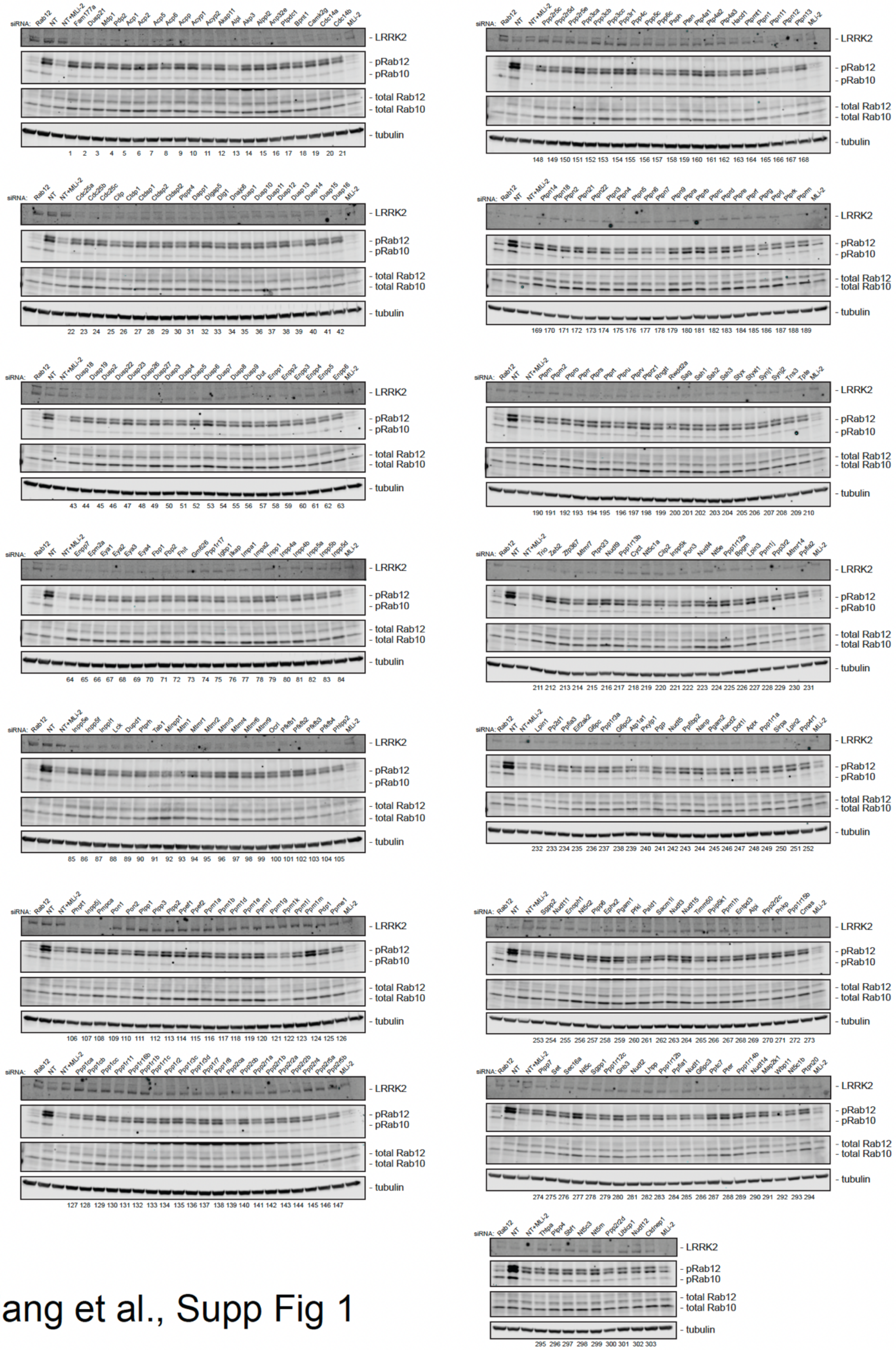

Chiang et al., Supp Fig 1

bioRxiv preprint doi: https://doi.org/10.1101/2025.03.19.644182; this version posted March 19, 2025. The copyright holder for this preprint (which was not certified by peer review) is the author/funder, who has granted bioRxiv a license to display the preprint in perpetuity. It is made available under aCC-BY 4.0 International license.

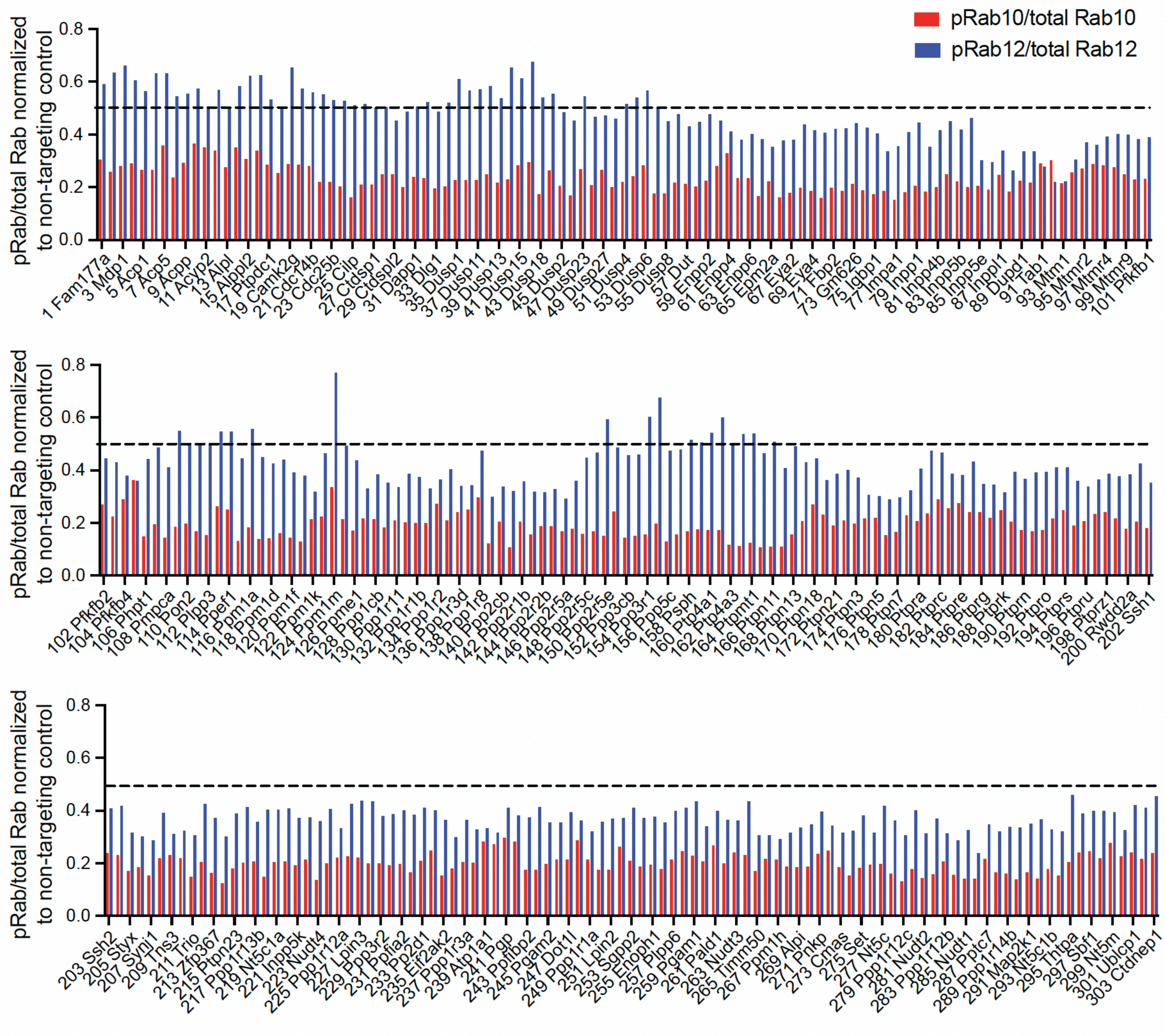

Chiang et al., Supp Fig 2

bioRxiv preprint doi: https://doi.org/10.1101/2025.03.19.644182; this version posted March 19, 2025. The copyright holder for this preprint (which was not certified by peer review) is the author/funder, who has granted bioRxiv a license to display the preprint in perpetuity. It is made available under aCC-BY 4.0 International license.

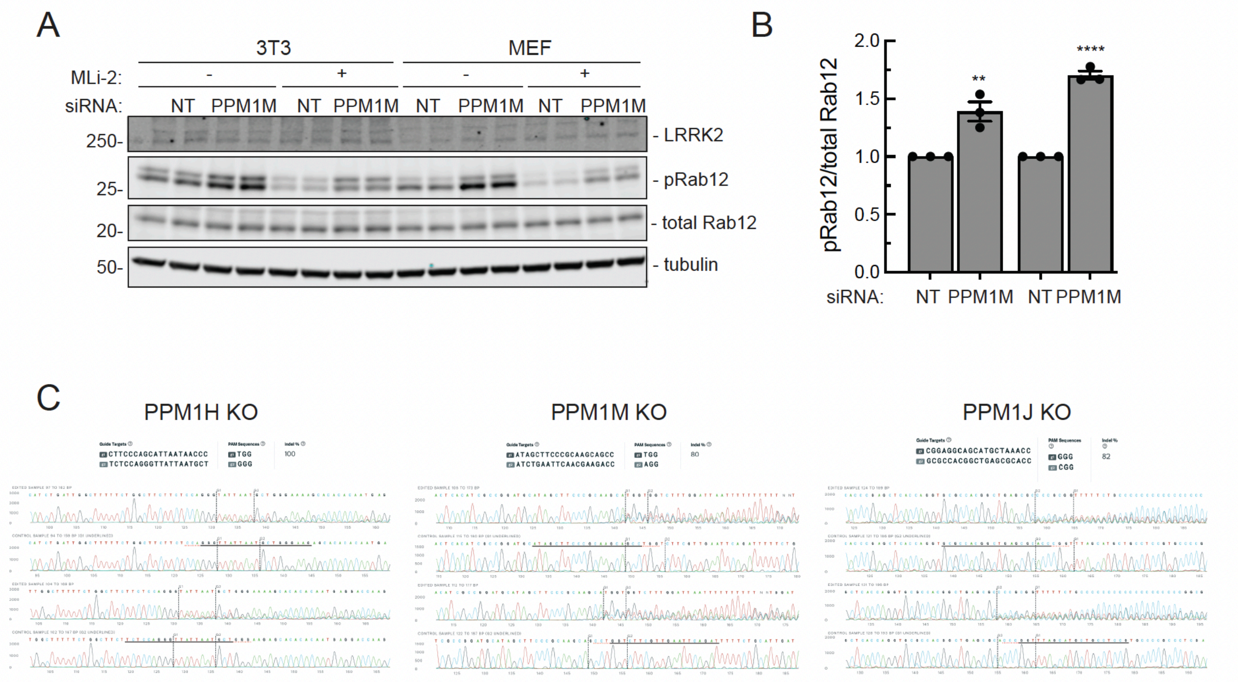

Chiang et al., Supp Fig 3

bioRxiv preprint doi: https://doi.org/10.1101/2025.03.19.644182; this version posted March 19, 2025. The copyright holder for this preprint (which was not certified by peer review) is the author/funder, who has granted bioRxiv a license to display the preprint in perpetuity. It is made available under aCC-BY 4.0 International license.

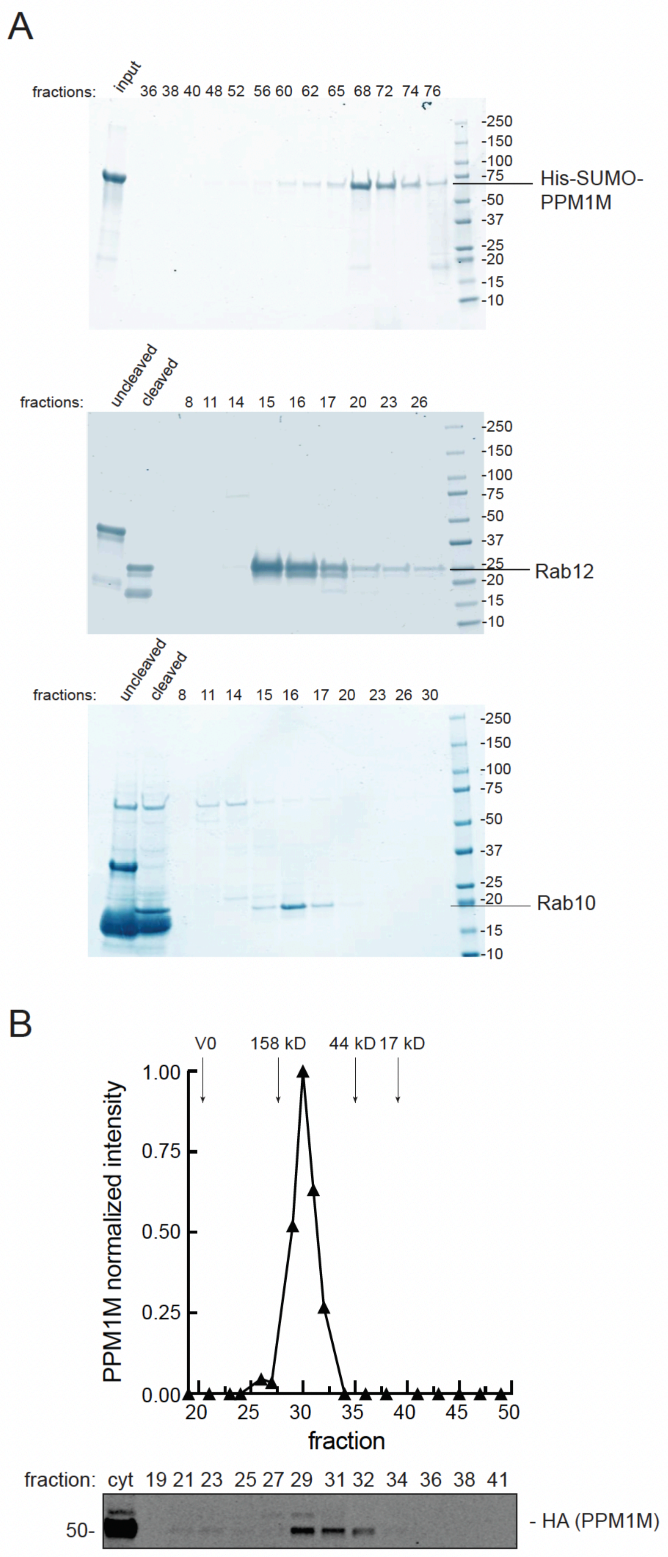

Chiang et al., Supp Fig 4

bioRxiv preprint doi: https://doi.org/10.1101/2025.03.19.644182; this version posted March 19, 2025. The copyright holder for this preprint (which was not certified by peer review) is the author/funder, who has granted bioRxiv a license to display the preprint in perpetuity. It is made available under aCC-BY 4.0 International license.

Supplemental Table 1. Key Resources Table

RESOURCE TYPE	RESOURCE NAME	SOURCE	IDENTIFIER	NEW/ REUSE	ADDITIONAL INFORMATION
Antibody	anti-GAPDH (mouse monoclonal)	Santa Cruz Biotechnology	sc-32233 (RRID: AB_627679)	REUSE	(1:5000)
Antibody	anti-PPM1H (rabbit monoclonal)	Abcam	ab303536 (RRID: AB_2941812)	REUSE	(1:2000)
Antibody	anti-alpha tubulin (mouse monoclonal)	Santa Cruz Biotechnology	sc-32293 (RRID: AB_628412)	REUSE	(1:4000)
Antibody	anti-HA (rabbit polyclonal)	Sigma	H6908 (RRID: AB_260070)	REUSE	(1:1000)
Antibody	anti-HA (mouse monoclonal)	Sigma	H9658 (RRID: AB_260092)	REUSE	(1:1000)
Antibody	anti-HA (rat monoclonal)	Sigma	11867423001 (RRID: AB_390918)	REUSE	(1:1000)
Antibody	anti-LRRK2 (mouse monoclonal)	Antibodies Incorporated/NeuroMab	N241A/34 (RRID: AB_10675136)	REUSE	(1:1000)
Antibody	anti-LRRK2 pS935 (rabbit monoclonal)	MRC PPU U. Dundee and/or Abcam Inc.	UDD2/ ab133450 (RRID: AB_2732035)	REUSE	(1:1000)
Antibody	anti-Rab10 pThr73 (rabbit monoclonal)	Abcam Inc.	ab230261 (RRID: AB_2811274)	REUSE	(1:1000)
Antibody	anti-Rab10 (mouse monoclonal)	Abcam Inc.	ab104859 (RRID: AB_10711207)	REUSE	(1:1000)
Antibody	anti-Rab12 pSer106 (rabbit monoclonal)	Abcam Inc.	ab256487 (RRID: AB_2884880)	REUSE	(1:1000)
Antibody	anti-Rab12 (mouse monoclonal)	Santa Cruz Biotechnology	sc-515613 (RRID: AB_3101762)	REUSE	(1:500)
Antibody	IRDye 800CW Donkey anti-Rabbit IgG	LI-COR	926-32213 (RRID: AB_621848)	REUSE	(1:10,000)
Antibody	IRDye 680RD Donkey anti-Rabbit IgG	LI-COR	926-68073 (RRID: AB_10954442)	REUSE	(1:10,000)
Antibody	IRDye 800CW Donkey anti-Mouse IgG	LI-COR	926-32212 (RRID: AB_621847)	REUSE	(1:10,000)
Antibody	IRDye 680RD Donkey anti-Mouse IgG	LI-COR	926-68072	REUSE	(1:10,000)

bioRxiv preprint doi: https://doi.org/10.1101/2025.03.19.644182; this version posted March 19, 2025. The copyright holder for this preprint (which was not certified by peer review) is the author/funder, who has granted bioRxiv a license to display the preprint in perpetuity. It is made available under aCC-BY 4.0 International license.

			(RRID: AB_10953628)		
Antibody	anti-Choline Acetyltransferase (goat polyclonal)	Sigma	208371 (RRID:AB_2079751)	REUSE	(1:200)
Antibody	anti-Adenylate cyclase III (rabbit polyclonal)	EnCOR	RPCA-ACIII (RRID:AB_2572219)	REUSE	(1:10,000)
Antibody	H+L Donkey anti-Goat AF 488	Life Technologies	A11055 (RRID:AB_2534102)	REUSE	(1:2000)
Antibody	H+L Donkey anti-Rabbit AF 568	Life Technologies	A10042 (RRID:AB_2534017)	REUSE	(1:2000)
Bacterial strain	MAX Efficiency™ DH5α Competent Cells	Invitrogen	18258012	REUSE	
Bacterial strain	BL21(DE3)pLysS	Novagen	69451-3	REUSE	
Chemical, peptide, or recombinant protein	MLi-2	MRC PPU Reagents and Services, U. Dundee	CAS No.: 1627091-47-7	REUSE	
Chemical, peptide, or recombinant protein	Instant Blue Coomassie	Abcam	ab119211	REUSE	
Chemical, peptide, or recombinant protein	Bio-Rad Protein Assay Dye Reagent Concentrate	Bio-Rad	5000006	REUSE	
Chemical, peptide, or recombinant protein	Dharmafect 1	Dharmacon	T-2001	REUSE	
Chemical, peptide, or recombinant protein	DMEM (high glucose)	Cytiva	SH30243.02	REUSE	
Chemical, peptide, or recombinant protein	Fetal bovine serum	Sigma	F0926	REUSE	

bioRxiv preprint doi: https://doi.org/10.1101/2025.03.19.644182; this version posted March 19, 2025. The copyright holder for this preprint (which was not certified by peer review) is the author/funder, who has granted bioRxiv a license to display the preprint in perpetuity. It is made available under aCC-BY 4.0 International license.

Chemical, peptide, or recombinant protein	Penicillin-Streptomycin	Sigma	P4333	REUSE	
Chemical, peptide, or recombinant protein	Opti-MEM	Gibco	31985088	REUSE	
Chemical, peptide, or recombinant protein	PEI, 25 kDa	Polysciences	23966	REUSE	
Chemical, peptide, or recombinant protein	cOmplete EDTA-free protease inhibitor cocktail	Roche	11873580001	REUSE	
Chemical, peptide, or recombinant protein	PhosSTOP phosphatase inhibitor cocktail	Roche	4906837001	REUSE	
Chemical, peptide, or recombinant protein	Microcystin-LR	Sigma	475815-M	REUSE	
Chemical, peptide, or recombinant protein	Mouse Rab12 siRNA (OnTarget, SMARTpool)	Dharmacon	L-040865-01	REUSE	
Chemical, peptide, or recombinant protein	Non-targeting siRNA (OnTarget, SMARTpool)	Dharmacon	D-001810-10	REUSE	
Chemical, peptide, or recombinant protein	Mouse phosphatase siRNA library (OnTarget, SMARTpool)	Dharmacon	G-113705, see Supplemental Table 2 for gene list	REUSE	
Chemical, peptide, or recombinant protein	Cherry-picked mouse phosphatase siRNA library (OnTarget, SMARTpool)	Dharmacon	custom, see Supplemental Table 2 for gene list	NEW	Additional phosphatase library genes not included in commercial phosphatase library
Chemical, peptide, or	GoTaq Green Master Mix	Promega	M7122	REUSE	

bioRxiv preprint doi: https://doi.org/10.1101/2025.03.19.644182; this version posted March 19, 2025. The copyright holder for this preprint (which was not certified by peer review) is the author/funder, who has granted bioRxiv a license to display the preprint in perpetuity. It is made available under aCC-BY 4.0 International license.

recombinant protein					
Critical commercial assay	4–20% Criterion TGX Precast Midi Protein Gel, 26 well, 15 µl	Bio-Rad	5671095	REUSE	
Critical commercial assay	Trans-Blot Turbo RTA Midi 0.2 µm Nitrocellulose Transfer Kit	Bio-Rad	1704271	REUSE	
Critical commercial assay	HiTrap TALON crude 1 mL column	Cytiva	28953766	REUSE	
Critical commercial assay	Econospin column	Epoch Lifesciences	1920-050/250	REUSE	
Critical commercial assay	Electroporation Cuvettes, 0.2 cm gap	Bio-Rad	1652086	REUSE	
Dataset	PPM1M, a LRRK2-counteracting, phosphoRab12-preferring phosphatase with potential link to Parkinson's disease	Zenodo	https://doi.org/10.5281/zenodo.14911979	NEW	raw tabular data and original tiff files for all western blot analysis, raw tabular data and original microscopy images for ciliation experiments
Experimental model: Cell line	HEK293T (human)	ATCC	CRL-3216 (RRID: CVCL_0063)	REUSE	
Experimental model: Cell line	A549 (human)	ATCC	CCL-185 (RRID: CVCL_0023)	REUSE	
Experimental model: Cell line	PPM1H knockout A549 (human)	MRC PPU Reagents and Services, U. Dundee	PMIID: 31663853	REUSE	
Experimental model: Cell line	Mouse Embryonic Fibroblasts (mouse)	MRC PPU Reagents and Services, U. Dundee	RRID: CVCL_E7DI	NEW	littermate match to PPM1M knockout MEFs
Experimental model: Cell line	PPM1M knockout Mouse Embryonic Fibroblasts (mouse)	MRC PPU Reagents and Services, U. Dundee	RRID: CVCL_E7DI	NEW	littermate match to WT MEFs

bioRxiv preprint doi: https://doi.org/10.1101/2025.03.19.644182; this version posted March 19, 2025. The copyright holder for this preprint (which was not certified by peer review) is the author/funder, who has granted bioRxiv a license to display the preprint in perpetuity. It is made available under aCC-BY 4.0 International license.

Experimental model: Cell line	3T3 Flp In (mouse)	Invitrogen	R76107 (RRID: CVCL_U422)	REUSE	
Protocol	Gibson Assembly	protocols.io	https://doi.org/10.17504/protocols.io.eq2lyjwyqlx9/v1	NEW	
Protocol	Phosphatome-wide siRNA screen in 3T3 cells	protocols.io	dx.doi.org/10.17504/protocols.io.36wgqdxr5vk5/v1	NEW	
Protocol	Creation of pooled CRISPR KO cell lines using Synthego sgRNA	protocols.io	dx.doi.org/10.17504/protocols.io.bp2l6dqodvqe/v1	REUSE	
Protocol	Isolation of mouse embryonic fibroblasts (MEFs) from mouse embryos at E12.5	protocols.io	dx.doi.org/10.17504/protocols.io.eq2ly713qlx9/v1	REUSE	
Protocol	Quantitative Immunoblotting Analysis of LRRK2 Signalling Pathway	protocols.io	dx.doi.org/10.17504/protocols.io.bsgrnbv6	REUSE	
Protocol	Expression and purification of PPM1H phosphatase	protocols.io	dx.doi.org/10.17504/protocols.io.bu7wnzpe	REUSE	
Protocol	Dephosphorylation of phosphorylated Rab GTPases by PPM phosphatases	protocols.io	dx.doi.org/10.17504/protocols.io.5jyl8d4j7g2w/v1	NEW	
Protocol	Crude Membrane Fractionation of Cultured Cells	protocols.io	https://doi.org/10.17504/protocols.io.yxmvmnb99g3p/v1	REUSE	
Protocol	Expression and purification of Rab8A (1-181) stoichiometrically phosphorylated at pThr72	protocols.io	dx.doi.org/10.17504/protocols.io.butinwke	REUSE	
Protocol	Analysis of Primary Cilia in Rodent Brain By Immunofluorescence Microscopy	protocols.io	dx.doi.org/10.17504/protocols.io.bnwimfce	REUSE	
Recombinant DNA	pCMV5D HA-PPM1H	MRC PPU Reagents and Services,	DU62789	REUSE	

bioRxiv preprint doi: https://doi.org/10.1101/2025.03.19.644182; this version posted March 19, 2025. The copyright holder for this preprint (which was not certified by peer review) is the author/funder, who has granted bioRxiv a license to display the preprint in perpetuity. It is made available under aCC-BY 4.0 International license.

		U. Dundee			
Recombinant DNA	pCMV5D HA-PPM1H H153D	MRC PPU Reagents and Services, U. Dundee	DU62928	REUSE	
Recombinant DNA	pCMV5D HA-PPM1H D288A	MRC PPU Reagents and Services, U. Dundee	DU62985	REUSE	
Recombinant DNA	pCMV5D HA-PPM1M	MRC PPU Reagents and Services, U. Dundee	DU68124	REUSE	
Recombinant DNA	pCMV5D HA-PPM1M H127D	MRC PPU Reagents and Services, U. Dundee	DU68165	REUSE	
Recombinant DNA	pCMV5D HA-PPM1M D235A	MRC PPU Reagents and Services, U. Dundee	DU68164	REUSE	
Recombinant DNA	pCMV5D HA-PPM1M D440N	MRC PPU Reagents and Services, U. Dundee	DU72159	NEW	
Recombinant DNA	pCMV5D HA-PPM1H_M flap	Addgene	Addgene in progress	NEW	
Recombinant DNA	pCMV5D HA-PPM1M_H flap	Addgene	Addgene in progress	NEW	
Recombinant DNA	Flag-LRRK2 R1441C	MRC PPU Reagents and Services, U. Dundee	DU13078	REUSE	
Recombinant DNA	Flag-LRRK2 R1441G	MRC PPU Reagents and Services, U. Dundee	DU26477	REUSE	
Recombinant DNA	His-SUMO-PPM1M	MRC PPU Reagents and Services, U. Dundee	DU68141	REUSE	

bioRxiv preprint doi: https://doi.org/10.1101/2025.03.19.644182; this version posted March 19, 2025. The copyright holder for this preprint (which was not certified by peer review) is the author/funder, who has granted bioRxiv a license to display the preprint in perpetuity. It is made available under aCC-BY 4.0 International license.

Recombinant DNA	His-SUMO-PPM1M H127D	MRC PPU Reagents and Services, U. Dundee	DU68200	REUSE	
Recombinant DNA	His-SUMO-PPM1M D440N	MRC PPU Reagents and Services, U. Dundee	DU72158	REUSE	
Recombinant DNA	His-SUMO-PPM1H	MRC PPU Reagents and Services, U. Dundee	DU62835	REUSE	
Recombinant DNA	His-SUMO-PPM1H D288A	MRC PPU Reagents and Services, U. Dundee	DU68087	REUSE	
Recombinant DNA	His-Thrombin-Rab8A (1-181)	MRC PPU Reagents and Services, U. Dundee	DU68198	REUSE	
Recombinant DNA	His-SUMO-Rab10 Q68L	Addgene	Addgene in progress	NEW	
Recombinant DNA	His-SUMO-Rab12 Q101L	Addgene	208371 (RRID:Addgene_208371)	REUSE	
Software/code	Alphafold Server	Alphafold 3	RRID: SCR_025885	REUSE	
Software/code	ChimeraX	ChimeraX	PMID: 32881101 (RRID: SCR_015872)	REUSE	
Software/code	ImageJ	ImageJ version 2.14	RRID: SCR_003070	REUSE	
Software/code	Graphpad Prism	Prism 10 version 10.2.3	RRID: SCR_002798	REUSE	
Software/code	ZEN	Zeiss ZEN Microscopy Software	RRID:SCR_013672	REUSE	

bioRxiv preprint doi: https://doi.org/10.1101/2025.03.19.644182; this version posted March 19, 2025. The copyright holder for this preprint (which was not certified by peer review) is the author/funder, who has granted bioRxiv a license to display the preprint in perpetuity. It is made available under aCC-BY 4.0 International license.

Supplemental Table 3: Overview of cohorts that were interrogated for *PPM1M* D440N carrier status

Study	N	Disease / control cohort (if applicable)	*PPM1M* D440N PD	*PPM1M* D440N non-PD	MAF
Hop et. al	71959	2184 (familial PD cases) / 69775 controls	3	3	3.81E-05
Austrian PD cohort	382	382 (familial and YOPD cases)	1	n.a.	-
Mayo clinic	700	700 (neuropathological specimens)	1	n.a.	-
Czechia PD cases	33	33	0	n.a.	-
Ireland PD cases	672	672	0	n.a.	-
Poland PD cases	725	725	1	n.a.	-
Ukraine PD cases	139	139	1	n.a.	-
gnomAD 4.1	800000	800000 (general population)	N/A	66	4.13E-05
Centogene	192000	10000 PD cases / 182000 non-PD cases	0	13	3.57E-05
